# Impact of vital signs screening & clinician prompting on alcohol and tobacco screening and intervention rates: a pre-post intervention comparison

**DOI:** 10.1186/1471-2296-11-18

**Published:** 2010-03-05

**Authors:** J Paul Seale, Sylvia Shellenberger, Mary M Velasquez, John M Boltri, Ike Okosun, Monique Guyinn, Dan Vinson, Monica Cornelius, J Aaron Johnson

**Affiliations:** 1 Department of Family Medicine, Medical Center of Central Georgia and Mercer University School of Medicine, 3780 Eisenhower Pkwy, Macon, GA 31206, USA; 2 School of Social Work, University of Texas-Austin, Austin, TX, USA; 3 Institute of Public Health, Georgia State University, Atlanta, GA 30302, USA; 4 Department of Family and Community Medicine, University of Missouri-Columbia, Columbia, MO 65212, USA

## Abstract

**Background:**

Though screening and intervention for alcohol and tobacco misuse are effective, primary care screening and intervention rates remain low. Previous studies have increased intervention rates using vital signs screening for tobacco misuse and clinician prompts for screen-positive patients for both alcohol and tobacco misuse. This pilot study's aims were: (1) To determine the feasibility of combined vital signs screening for tobacco and alcohol misuse, (2) To assess the impact of vital signs screening on alcohol and tobacco screening and intervention rates, and (3) To assess the additional impact of tobacco assessment prompts on intervention rates.

**Methods:**

In five outpatient practices, nurses measuring vital signs were trained to routinely ask a single tobacco question, a prescreening question that identified current drinkers, and the single alcohol screening question for current drinkers. After 4-8 weeks, clinicians were trained in tobacco intervention and nurses were trained to give tobacco abusers a tobacco questionnaire which also served as a clinician intervention prompt. Screening and intervention rates were measured using patient exit interviews (n = 622) at baseline, during the "screening only" period, and during the tobacco prompting phase. Changes in screening and intervention rates were compared using chi square analyses and test of linear trends. Clinic staff were interviewed regarding patient and staff acceptability. Logistic regression was used to evaluate the impact of nurse screening on clinician intervention, the impact of alcohol intervention on concurrent tobacco intervention, and the impact of tobacco intervention on concurrent alcohol intervention.

**Results:**

Alcohol and tobacco screening rates and alcohol intervention rates increased after implementing vital signs screening (p < .05). During the tobacco prompting phase, clinician intervention rates increased significantly for both alcohol (12.4%, p < .001) and tobacco (47.4%, p = .042). Screening by nurses was associated with clinician advice to reduce alcohol use (OR 13.1; 95% CI 6.2-27.6) and tobacco use (OR 2.6; 95% CI 1.3-5.2). Acceptability was high with nurses and patients.

**Conclusions:**

Vital signs screening can be incorporated in primary care and increases alcohol screening and intervention rates. Tobacco assessment prompts increase both alcohol and tobacco interventions. These simple interventions show promise for dissemination in primary care settings.

## Background

One in three adults worldwide is a regular smoker, and among these adults, 50% will die from cigarettes [1]. Cigarette smoking is the most important source of preventable morbidity and mortality in the US, with cost exceeding $167 billion annually [2]. Almost 30% of primary care patients use some form of tobacco [3].

Alcohol use is related to a wide range of harms [4]. Approximately one-third of all US adults are involved in alcohol misuse [5], including 14 million adults with alcohol abuse or alcoholism and almost twice that number who are involved in at-risk drinking (for women, more than three drinks per day or seven drinks per week and for men, more than four drinks per day or 14 drinks per week). The cost of alcohol misuse in the U.S. is approximately $140 billion annually [6]. Among primary health care patients, 7 to 20 percent engage in alcohol misuse [7]. Alcohol use and tobacco use are often associated, with approximately 50% of alcohol misusers concurrently using tobacco [8,9]. Concurrent tobacco and alcohol use synergistically increase the risk of head and neck and esophageal cancer [10,11]. Binge drinking and alcohol abuse also hinder individuals' efforts to stop smoking [12-14].

Primary care intervention for alcohol and tobacco misuse is effective and reduces morbidity and mortality. Brief advice by primary care clinicians increases quit rates for smokers [15,16]and helps hazardous and harmful drinkers decrease their alcohol consumption [17]. Smoking cessation significantly reduces future cancer risk [18-22]. The U.S. Preventive Services Task Force now recommends screening and office intervention for both tobacco use and problem drinking [23,24]. Combined tobacco and alcohol intervention has the potential to reduce tobacco- and alcohol-related morbidity and cancer risk. Nonetheless, tobacco and alcohol screening and intervention are not consistently performed in most primary care practices. Despite education and training initiatives in alcohol [25-27] and tobacco [1,8,28-30], health care providers screen only 28% of patients for alcohol misuse [31] and 48% for tobacco abuse [1,28,32,33].

In an effort to increase screening rates, office protocols have been developed to screen patients before they see their clinicians and prompt clinicians to intervene when screens are positive. Using a vital signs stamp that assesses smoking status as part of nursing vital signs has been shown to increase tobacco cessation rates in three of four studies [34-37]. For alcohol screening, a validated single alcohol screening question (SASQ: "When was the last time you had more than X drinks in one day?" where X = 4 for women and 5 for men) has demonstrated acceptable sensitivity and specificity [8,38,39], however no previous studies have attempted to include this question in nursing vital signs.

Most studies employing reminder systems which prompt primary care clinicians to intervene have resulted in increased clinician intervention rates for tobacco abuse [40-42] and alcohol misuse [43-45], though some studies have showed mixed results [27]. A study by Milch et al [32] combined vital signs screening with nurse administration of a brief tobacco assessment instrument which was then used as a clinician prompt, resulting in increased tobacco interventions by clinicians and higher rates of self-reported smoking cessation. Findings from this single study, however, have not yet been replicated. The purposes of this multi-site study were to: (1) determine the feasibility of combined vital signs screening for tobacco and alcohol misuse, (2) assess the impact of vital signs screening alone on alcohol and tobacco screening and intervention rates, and (3) assess the impact of combined vital signs screening plus use of a tobacco prompt for increasing tobacco interventions.

## Methods

### Study Site Recruitment

This study was approved by the Institutional Review Board of the Medical Center of Central Georgia in Macon, GA, and was conducted from July 2004 to July 2005. A total of 625 patients were eligible and consented to participate in this study.

Interviews were conducted at five Central Georgia family physician offices within the Georgia-Mercer Primary Care Research Network. All clinicians at these offices (physicians, nurse practitioners, and physician assistants) were chosen because of their participation in previous research network studies and were recruited by personal interview. Participation rate was 100% (no refusals). All five were single specialty offices, and two offices had physician extenders (one nurse practitioner and one physician assistant). Typical of family physicians, all five clinician offices treat the full range of patients: infants to the elderly, with a wide range of problems. The number of patient visits per day ranged from 20 to 60 per clinician. There was a mix of fee for service, HMO, PPO, Medicare and Medicaid patients at each office.

### Study Design

**Figure 1:**
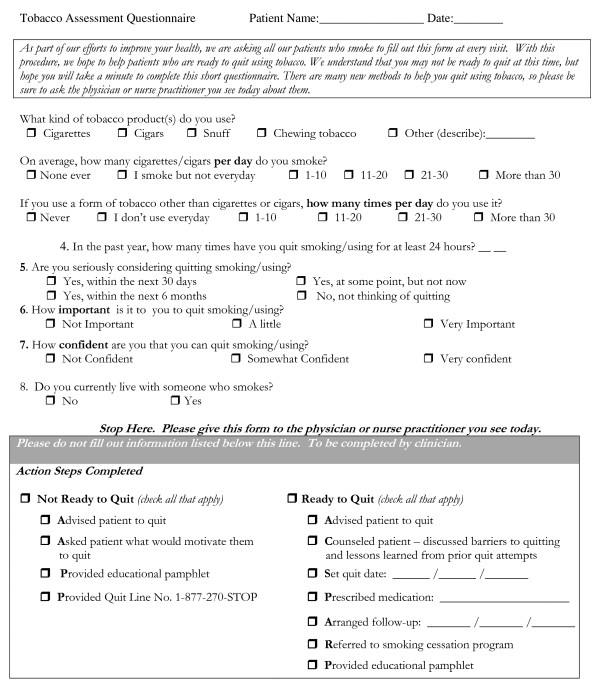
**Tobacco Assessment Questionnaire**.

This was a pre-post intervention study conducted in three phases of approximately 50 days each. Prior to integrating alcohol and tobacco vital signs screening into the clinic protocol, baseline alcohol and tobacco screening and intervention rates were collected in Phase I. The effect of each of the study's two interventions was measured in phases II and III. Vital signs screening only was implemented in Phase II, followed by vital signs screening plus the tobacco prompt in Phase III. Changes in the following variables were assessed across the study's three phases: alcohol screening by nurses, alcohol screening by clinicians, alcohol screening by nurses and/or clinicians, clinician alcohol intervention, tobacco screening by nurses, tobacco screening by clinicians, tobacco screening by nurses and/or clinicians, and clinician tobacco interventions. For patients reporting tobacco interventions, we also measured the frequency with which clinicians performed any of the seven steps listed on the tobacco intervention prompt (see Figure 1).

**Figure 2:**
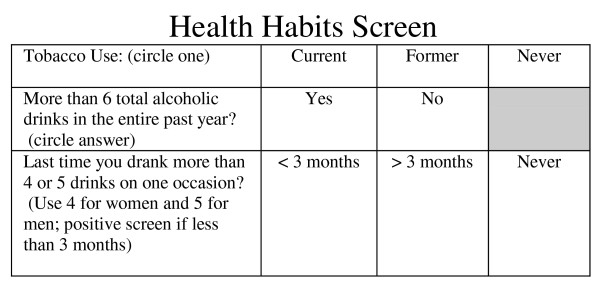
**Health Habits Stamp for Vital Signs Screening**.

In each phase, approximately 40 patients per practice completed exit interviews. Prior to baseline measurement, clinicians were informed that the study would involve testing simple interventions that nurses might perform as part of patient vital signs that might increase tobacco and alcohol screening and intervention. Clinicians were not informed in advance of patient interview days but were often aware when the interviewer was present. In Phase II, which assessed the impact of including alcohol and tobacco screening questions in the nursing vital signs, screening was performed using three questions. The first assessed tobacco use. The second question ("Have you had more than six alcoholic drinks in the past year?") identified patients who were current drinkers. The third question, the SASQ was administered only to patients identified as current drinkers. A vital signs "stamp" was created which could be used to stamp these three questions onto the vital signs portion of the patient's chart (see Figure 2). A fifteen-minute orientation session was conducted to orient clinicians to the project procedures and train nurses to ask the three questions as part of nursing vital signs. Procedures were modified for two clinics with electronic medical records which used templates that could not easily be modified to include the screening questions as part of nursing vital signs. In one clinic, the screening questions were printed on sheets of paper and placed in each examining room on clipboards. Nurses were instructed to ask the questions when they placed patients in the room. In the other clinic, the questions were stamped on the paper where nurses routinely recorded each patient's vital signs and left them in the exam room for the physician. Clinicians were informed that positive screens included any tobacco use or drinking more than four (for females) or five drinks (for males) on any day in the past three months, and were encouraged to talk with patients about modifying these habits. In Phase III, vital signs screening continued, and nurses were asked to give a tobacco assessment instrument, adapted from Milch et al [32], to all patients reporting tobacco use (see Figure 1), ask them to complete it and give it to their clinician. All clinicians also received one hour of tobacco intervention training using the steps of the Agency for Healthcare Research and Quality's Clinician's Guide [46]. No specific training was provided regarding further alcohol assessment or brief intervention.

The feasibility of vital signs screening was assessed by interviewing nurses and clinicians and by observation of nurses and clinician behavior by two project investigators (JPS and MG) during Phases II and III at each clinic. The impact of the Phase II and III interventions on alcohol screening and intervention rates was assessed via patient exit interviews.

### Patient Recruitment and Eligibility Criteria

In order to monitor clinic screening and intervention rates, patients were recruited in clinic waiting areas and written informed consent was obtained. Each male patient and every other female patient was invited to participate. Patients first completed a four-question written health habits questionnaire regarding weight, consumption of saturated fats, tobacco use, and alcohol use. English-speaking patients who had consumed at least six alcoholic drinks in the past year and had not already been interviewed for this study met inclusion criteria for the study and were asked to return for an interview after seeing their primary care clinician.

### Measures

The exit interview included demographic information including ethnicity, which was recorded based on patients' self-report. The interview also included the Diagnostic Interview Schedule for alcohol use disorders [47] and a 29-day alcohol Timeline Followback Interview [48]. Alcohol screening was defined as an affirmative answer to the question, "Today, did the nurse who took your blood pressure ask you about your alcohol use?" Alcohol intervention was defined as an affirmative answer to the question, "Today, did the doctor discuss cutting down or stopping your alcohol use?" Similarly worded questions were used to define tobacco use and intervention. Patients who reported being advised to reduce their tobacco use were asked whether the clinician had encouraged them to set a quit date, discussed possible use of pharmacotherapy, given them a smoking cessation handout, or given them information regarding tobacco cessation programs or telephone counseling services.

To determine the percentage of patients who should receive an intervention we calculated the prevalence of alcohol misuse and tobacco abuse using exit interview responses. Alcohol misuse was defined as presence of a DSM-IV [49] alcohol use disorder or at-risk drinking, defined using NIAAA criteria of more than three drinks in a day or seven drinks in a week for women and more than four drinks in a day or 14 drinks in a week for men [50]. Tobacco abuse was defined as any self-reported tobacco use. All patients with alcohol misuse or tobacco abuse were considered as appropriate patients for clinician interventions.

### Statistical Analyses

Statistical programs that are available in SPSS for Windows (version 14.0) were used for analysis [51]. Ethnic differences in age stratified by gender were determined using independent t-tests. Ethnic specific rates for alcohol misuse and tobacco abuse were also stratified by gender, and differences in rates were determined using chi-square tests. Changes in screening and intervention rates across study phases were determined using tests of linear trends. Pair differences in screening rates between individual study phases were measured using chi-square tests.

To assess the odds of alcohol and tobacco interventions that were associated with nurse screening (independent variable), we fitted both univariate and multivariate logistic regression models using alcohol intervention and tobacco intervention as dependent variables. We compared odds ratios of alcohol intervention and tobacco intervention that were associated with nurse screening as part of vital signs. To investigate whether offering tobacco advice was associated with offering alcohol advice and vice versa, we also fitted univariate and multivariate logistic regression models in which first alcohol advice, then tobacco advice was used as the dependent variable. In all multivariate analyses, statistical adjustments were made for patient age, gender and ethnicity. The customary p-values of < 0.05 and 95% confidence intervals were used to indicate statistical significance.

## Results

Vital signs screening was successfully implemented during a two-month period at all clinics. Although some nursing staff expressed concerns about asking all patients about the sensitive subject of alcohol prior to startup, these concerns dissipated during the first week as they quickly adapted to talking with patients about their tobacco and alcohol use. After implementation, nurses reported that patients readily accepted the vital signs screening as a part of their visit and that most answered questions freely, with few questions or objections. Two clinics with electronic medical records were unable to insert the alcohol and drug questions into their vital signs templates and collected this information using paper sheets. This was problematic in one clinic, as this created a new procedure which required nurses to remember to pick up a clipboard and ask the screening questions. In the other clinic, fewer problems were experienced: the receptionist stamped the vital signs questions onto the paper where nurses routinely recorded vital signs, thereby integrating the new questions into the existing vital signs procedure.

A total of 622 patients (99.5% of patients who consented to participate) provided complete answers to all questions. Approximately 120 patients were recruited from each of the participating five primary care practices. Subject refusal rate, measured during the final month of the study, was 17.5%.

Of the study participants, 378 (60.7%) were white and 238 (38.2%) were African American. Six (1%) were of other races. Mean (SD) age was 40.4 (12.8) years (median = 40) for women and 42.1 (13.7) years (median = 41) for men. Age and gender distribution of subjects is shown in Table 1. Among men, more whites than African Americans met diagnostic criteria for alcohol misuse (p < .05), however tobacco abuse rates showed no significant differences. There were no statistically significant ethnic differences in rates of alcohol misuse or tobacco abuse among women.

**Table 1:** Characteristics of Study Population

	Men			Women		
	White	Black	*p*	White	Black	*p*
	(n = 189)	(n = 92)		(n = 189)	(n = 146)	
Age, in years						
Mean (SD)	42.7 (14.3)	41.0 (12.6)	.388	40.4 (13.5)	40.5 (11.8)	.992
Median	42	41.5		40	41	
Alcohol misuse	45.2%	31.5%	.029	29.6%	30.8%	.814
Tobacco abuse	41.8%	33.0%	.156	39.2%	37.7%	.782

*Notes*: Alcohol misuse is defined as at-risk drinking (consumption of more than 3 drinks in 1 day or 7 in 1 week for women, and more than 4 in 1 day or 14 in 1 week for men) and/or presence of a DSM-4 alcohol-use disorder (alcohol abuse and/or alcohol dependence).

Low rates of alcohol screening were reported during the baseline period for both nurses alone and for nurses and clinicians combined (9.5 and 14.7% of patients, respectively; see Table 2). Both these rates increased significantly after implementing vital signs screening (38.1% and 40.5%, respectively) in Phase II, then plateaued in Phase III, with overall screening rates remaining near 40%. Alcohol screening and alcohol intervention by clinicians increased modestly but significantly across all phases (p < .001). Changes in tobacco screening rates followed a similar pattern to those observed with alcohol. Tobacco screening rates for nurses alone and for nurses and clinicians combined increased significantly from baseline (20.4% and 28.0%, respectively) to Phase II (44.8% and 49.5%, respectively), then plateaued, with overall screening rates remaining near 50%. Interestingly, tobacco intervention rates by clinicians ("clinician advice to quit") showed no increase after implementation of vital signs screening alone (Phase II), then increased significantly with the addition of clinician prompting in Phase III to 47.4% (p = .042). Statistical analysis also revealed significant increases across study phases in three individual quit steps (asking patients what would motivate them to quit, giving pamphlets, and discussions about quit dates) and highly significant increases in two steps: discussing quit medications and prescribing quit medications.

**Table 2:** Changes in Alcohol and Tobacco Screening and Intervention Rates by Study Phases (%)

	Phase I	Phase II	Phase III	*p*
	(n = 211)	(n = 210)	(n = 201)	
**Alcohol Screening and Intervention Rates**				
Screening by Nurses	**9.5**^**a**^	**38.1**^**b**^	**32.3**^**c**^	**.018**
Screening by Clinicians	**9.5**^**a**^	**13.4**^**b**^	**21.9**^**c**^	**<.001**
Any Screening (Nurses or Clinicians)	**14.7**^**a**^	**40.5**^**b**^	**37.8**^**c**^	**.041**
Clinician Alcohol Interventions	**3.8**^**a**^	**6.7**^**b**^	**12.4**^**c**^	**<.001**
				
**Tobacco Screening Rates**				
Screening by Nurses	**20.4**^**a**^	**44.8**^**b**^	**41.3**^**c**^	**.081**
Screening by Clinicians	**18.5**	**18.1**	**26.0**	**.121**
Any Screening (Nurses or Clinicians)	**28.0**^**a**^	**49.5**^**b**^	**49.0**^**b**^	**.371**
				
**Tobacco Intervention Rates**	**(n = 87)**	**(n = 76)**	**(n = 76)**	
Clinician advice to quit	**32.2**^**a**^	**30.3**^**b**^	**47.4**^**c**^	**.042**
Specific Tobacco Quit Steps				
Asked what would motivate patient to quit	**14.3**	**17.6**	**25.0**	**.021**
Gave patient a pamphlet	**9.1**	**9.5**	**15.4**	**.017**
Discussed lessons learned from past attempts	**7.9**	**6.8**	**9.2**	**.421**
Discussed setting a date to quit	**3.9**	**8.1**	**10.8**	**.029**
Discussed nicotine products or other meds	**9.2**^**a**^	**16.2**^**b**^	**24.2**^**c**^	**<.001**
Prescribed bupropion or a nicotine product	**3.9**^**a**^	**5.4**^**b**^	**12.1**^**c**^	**<.001**
Gave info about Tobacco Quit Line	**5.3**	**1.4**	**6.1**	**.711**
Referred to a smoking cessation program	**2.6**	**2.7**	**3.0**	**.052**

Values with different superscripts differ from each other at p < .05 on pairwise comparison; p-values listed in the table arefrom test of linear trends across study phases

Logistic regression analysis demonstrated that nurse screening significantly increased the odds of alcohol intervention, both before adjusting for sociodemographic variables (odds ratio [OR] 12.9, 95% CI: 6.3 - 26.7) and after adjusting (OR 13.1, 95% CI 6.2-27.6). Nurse screening also significantly improved the odds of tobacco intervention both before (OR 2.1, 95% CI 1.1-5.9) and after adjusting for demographic variables (OR 2.6, 95% CI: 1.3 - 5.2). Giving tobacco advice was associated with increased odds of giving alcohol advice in both univariate (OR 6.8, 95% CI 2.56-17.8) and multivariate (OR 7.4; 95% CI 2.8-20.1) analyses. Similarly, giving alcohol advice was also associated with increased odds of giving tobacco advice both before (OR 6.89: 95% CI 3.14-15.15) and after adjusting for age, gender and ethnicity (OR 7.47: 95% CI 3.32-16.79).

## Discussion

Previous primary care studies have used health screening surveys to assess alcohol and tobacco use [52-54], but the current study is one of the first to incorporate screening for both alcohol and tobacco as part of the vital signs. Screening was accomplished using the SASQ [39], which was easily included in the brief nursing encounter used for vital signs measurement. The three-question screening system designed for this study proved to be acceptable to nurses, clinicians and patients and resulted in significant increases in alcohol and tobacco screening. Although universal screening was not achieved, alcohol screening rates were similar to the 44-50% range seen in previous multi-site studies designed to increase alcohol screening in primary care [44,45,55]. Results from Phase II of this study demonstrated modest but significant increases in clinician interventions for alcohol but not for tobacco from vital signs screening alone. Our findings are similar to those of Piper et al [37], who found no increase in tobacco intervention rates from vital signs screening for tobacco alone.

This study's more intensive intervention, the addition of a previously-studied tobacco assessment instrument which was also used as a clinician prompt, further improved rates of clinician tobacco cessation advice. Clinician prompts have previously been shown to increase clinician interventions with other health habits such as alcohol misuse [44,45]. Our study confirms previous findings of Milch et al [32] regarding the effectiveness of a tobacco prompt in increasing tobacco intervention rates. In this study, clinician cessation advice rates attained with use of the prompt (47%) were equal to those found in Milch's study. There were also significant increases in multiple tobacco cessation counseling steps advocated in the steps of the Agency for Healthcare Research and Quality's Clinician's Guide [46] such as setting a quit date and exploring patients' motivation for quitting, indicating that clinicians went beyond simple brief advice. The most significant increases were seen in clinicians' discussing and prescribing quit products, which have been proven to increase patient abstinence rates [56].

This study addresses an important current need in primary care: the development of efficient methods for addressing multiple health risk behaviors in a single visit [57,58]. Existing research indicates that physicians limit the preventive services they offer due to time constraints and the high number of competing demands encountered during a single office visit [59,60]. Physicians also report a lack of confidence in their ability to screen and provide brief advice [61]. A somewhat surprising finding of this study was the fact that vital signs screening for both tobacco and alcohol misuse, followed by clinician prompts for tobacco abuse only, resulted in increases in both alcohol and tobacco interventions. Despite the time constraints, clinicians in this study who dedicated time to tobacco assessment and counseling were also more likely to perform alcohol interventions. This finding suggests that clinicians providing advice to change one lifestyle behavior may find it easier to address a second behavior. Starting with tobacco use as the target behavior might also increase the physician's confidence in providing screening and advice for alcohol misuse. Further study is needed to explore the link between advice for these two behaviors and perhaps better utilize tobacco cessation encounters as an opportunity to increase clinician alcohol intervention rates.

### Limitations

This study, performed in five single physician practices in the southeastern U.S., may not be representative of other areas of the U.S. However, two strengths of the study are the ethnic diversity of the patient population studied and the involvement of multiple practice sites. Data regarding tobacco screening and intervention were collected only on current drinkers and may not be representative of all tobacco users. Lack of blinding physicians to the presence of the patient interviewer may have influenced their clinical behavior. This study, which was designed to assess practice change behavior, did not assess the impact of clinician interventions on patients' subsequent alcohol and tobacco use or solicit patient feedback regarding the vital sign screening process.

## Conclusions

This study demonstrates that vital signs screening is a simple, efficient and well-accepted method for conducting alcohol and tobacco screening in primary care, and that this intervention, when combined with clinician prompting, increases alcohol and tobacco screening and intervention rates. Importantly, the tobacco assessment prompts in this study increased both alcohol and tobacco interventions. While this simple, low-cost intervention resulted in modest but significant increases, future research should explore interventions which would further increase these rates. Techniques which have been utilized in other studies include emphasizing the link between alcohol misuse and biomedical consequences such as hypertension [62] or abnormal laboratory tests [63], academic detailing [64], and use of a more complex systems-based quality improvement intervention approach [65]. Future studies might also include training in alcohol intervention, which has been shown to increase clinician confidence in performing brief intervention [66-69], and test use of a combined sheet with both tobacco and alcohol brief advice steps, in order to improve translation of effective intervention strategies for alcohol and tobacco in primary care.

## Competing interests

The authors declare that they have no competing interests.

## Authors' contributions

JPS is the corresponding author and created the study design, methods, analysis and participated in manuscript preparation. SS participated in the creation of the study design, analysis, discussion and manuscript preparation. MMV participated in the creation of the analysis, discussion and manuscript preparation. JMB participated in the creation of the study design, discussion and manuscript preparation. IO participated in statistical analysis, conclusions and manuscript preparation. MG participated in data collection, analysis and discussion. DV participated in statistical analysis, discussion and conclusions. MC participated in data collection, statistical analysis, and discussion. JAJ participated in statistical analysis, discussion and conclusions. All authors have read and approved the final manuscript.

## Pre-publication history

The pre-publication history for this paper can be accessed here:

http://www.biomedcentral.com/1471-2296/11/18/prepub
